# Impact of confounding by smoking on cancer risk estimates in cohort studies of radiation workers: a simulation study

**DOI:** 10.1093/jrr/rraf012

**Published:** 2025-03-20

**Authors:** Kazutaka Doi, Shinji Yoshinaga

**Affiliations:** Department of Radiation Regulatory Science Research, Institute for Radiological Science, National Institutes for Quantum Science and Technology, 4-9-1, Anagawa, Inage-Ku, Chiba City 263-8555, Japan; Department of Environmetrics and Biometrics, Research Institute for Radiation Biology and Medicine, Hiroshima University, 1-2-3 Kasumi, Minami-Ku, Hiroshima City 732-8553, Japan

**Keywords:** confounding, cohort study, smoking, radiation workers, simulation study, cancer risk estimate

## Abstract

Previous studies on cohorts of radiation workers have provided valuable insights into the effects of low-dose-rate radiation; however, some concerns regarding the potential confounding effects of smoking have been expressed. Although some studies have collected smoking data and adjusted for this variable, their limited numbers and the presence of other confounders obscure the extent of the impact of smoking on their results. To address this, we conducted a simulation study to quantitatively evaluate the bias from confounding and modeling conditions, similar to actual epidemiological studies. Our analysis, based on data from Japanese radiation workers, indicated that not adjusting for smoking can lead to an overestimation of radiation effects by approximately 110%. This overestimation was relatively insensitive to sample size and dose distribution parameters, but varied with radiation and smoking risk, baseline smoking probability, and heterogeneity in baseline risk. Considering the simplified settings of this simulation study and the uncertainty of the estimates of Japanese radiation workers, our simulation results were consistent with those of the real-world epidemiological study. We also compared the results using Cox and Poisson regression models, ensuring that their modeling approaches were as similar as possible, and found minimal differences between them.

## INTRODUCTION

To evaluate the cancer risks associated with long-term exposure to low-dose-rate radiation, numerous cohort studies have focused on radiation workers [1–3]. Epidemiological studies have been conducted on radiation workers in various countries, with some of them combining data from multiple national cohorts [4, 5].

However, most of these studies did not sufficiently adjust for smoking, which may have distorted their assessments of the relationship between radiation exposure and cancer-associated mortality. As a result, there are concerns that current estimates of radiation risk may be biased by the confounding influence of smoking. Although some studies have attempted to indirectly evaluate the impact of smoking by treating cancers that are typically related to smoking as censored cases, it remains unclear whether these efforts adequately adjusted for its effects [4].

Stratification and regression models using relevant factors are common methods used to adjust for confounding factors in epidemiology. Some studies on radiation workers have collected smoking information and attempted to adjust for this confounding factor by including smoking as a covariate in their analyses [6]. However, such studies are limited and may have been confounded by other factors as well. The extent to which smoking-induced confounders affect cancer risk estimates therefore remains poorly understood in the current literature.

Therefore, a simulation study was conducted to quantitatively evaluate the degree of bias introduced by confounding factors, mimicking the conditions of actual epidemiological studies. In this cohort study, we investigated cancer-associated mortality among radiation workers and generated follow-up information as a simulation dataset. By analyzing this dataset to estimate cancer risk, we assessed the impact on risk estimates when confounding by smoking was not adequately adjusted for.

## MATERIALS AND METHODS

### Cohort study assumed in data generation

This prospective cohort study investigated cancer-related deaths among male radiation workers. The default cohort size was 80 000 individuals because it matched the number of participants in one cohort study of radiation workers in Japan (J-EPISODE) [6]. However, we also considered sample sizes of 20 000, 40 000, and 200 000 for comparison. Participants entered the cohort at 20 years of age and were followed up for 60 years, ending at 80 years of age or upon the occurrence of an event or censoring. The participants either died from cancer before the end of the follow-up period, died from other causes, or were observed until the end of the follow-up period. Death from cancer was considered an event, whereas all other outcomes were treated as censoring.

### Cancer and noncancer-associated mortality

To determine the risk of cancer-associated mortality, we used data concerning cancer-associated mortality by sex from the 2016 Vital Statistics in Japan database [7]. As cancer-related death hazards can be modeled using a Weibull distribution, we generated the event times for cancer-related deaths as Weibull random numbers. The shape parameter $a$ and scale parameter $b$ of the Weibull distribution were set to $\left(6.086609,75.992411\right)$ to closely match the Vital Statistics data. Cancer death times were generated as Weibull random numbers with parameters $\left(a,b/\left({\lambda}^{1/a}\right)\right)$ to reflect the hazard ratio $\lambda$, which increases with both radiation dose and smoking.

For non-cancer-associated mortality, we calculated the hazard ratio by excluding cancer-related deaths from all deaths according to sex. Because it was difficult to express non-cancer-associated mortality using a Weibull distribution, the hazard function was modeled as a quadratic function of age *t*, with age 20 set as zero: $\exp \left(0.001022\ast{\left(t+0.05311\right)}^2-7.221\right)$. Random numbers following this hazard were generated using the inversion method [8].

However, generating random numbers with this approach was several hundred times slower compared to that with direct generation, such as for Weibull random numbers. As a result, data assumed to follow a Weibull distribution were generated directly as Weibull random numbers, and the inversion method was employed only when the data could not be assumed to follow a Weibull distribution.

### Radiation and smoking data

The participants had received all radiation doses by the start of the follow-up period, and their smoking statuses remained constant throughout it.

The radiation dose for each participant was assumed to follow a log-normal distribution, generated as a log-normal random number. The geometric mean and standard deviation and the parameters of the log-normal distribution were set to 0.2623 and 2.223, respectively, based on a previous epidemiological study in Japan [9]. Doses exceeding 1 Gy were reduced to 1 Gy.

The smoking data were generated as a binary variable. Based on the National Health and Nutrition Survey [10], the probability of smoking for a participant with 0 Gy exposure was set to 0.3, with the probability increasing along with increasing radiation doses. The odds for the probability of smoking at 1 Gy were set to 0.1, 0.5, 1.0, 2.0, 4.0, 8.0, 16.0 and 32.0.

### Risk models for radiation and smoking

Epidemiological studies on radiation often present risk estimates as excess relative risk (ERR). However, when the sample size is small, ERR estimates have a skewed distribution that requires correction [11]. Methods have been proposed to correct this bias, particularly in case–control studies; however, these approaches have several drawbacks—including limited applicable models, increased computation time, and convergence issues during parameter estimations [12, 13]. To avoid these complications and focus on the effects of confounding factors, this study modeled the risk of radiation and smoking as a hazard ratio. The hazard for the *i*-th participant at time *t* was modeled as ${\lambda}_i(t)={\lambda}_0(t)\exp \left({\mathrm{\beta}}_r{dose}_i+{\mathrm{\beta}}_s{smk}_i+{\mathrm{\beta}}_u{u}_i\right)$, where ${dose}_i$, ${smk}_i$ and ${u}_i$ represent the radiation dose, smoking and unmeasured status of the participant, and ${\mathrm{\beta}}_r$, ${\mathrm{\beta}}_s$, and ${\mathrm{\beta}}_u$ are the parameters for radiation, smoking and unmeasured status, respectively. ERR was defined as relative risk minus 1 [14]; the corresponding ERR could then be calculated by subtracting 1 from the hazard ratio. Unmeasured state variables ${u}_i$ were generated as variables following a standard normal distribution to examine the impact of baseline risk heterogeneity on the results.

The hazard ratio for cancer deaths at 1 Gy relative to 0 Gy was set to 1.3 by default (equivalent to an ERR of 0.3) based on a previous epidemiological study in Japan [6] and varied across values of 1.1, 2.0, 5.0 and 10.0, corresponding to ERR values of 0.1, 1.0, 4.0 and 9.0, respectively. No modifying effects of age at exposure or attained age were assumed. The hazard ratio due to smoking was set to 1.6 [15] and varied across values of 1.1, 2.0, 5.0 and 10.0. The coefficient ${\mathrm{\beta}}_u$ for the unmeasured state variables was set to 0 by default and was varied across values of 0.1, 0.5 and 1.0.

As addition within the exponential function corresponded to multiplication outside the function, this model was multiplicative for radiation and smoking. In the life span study of atomic bomb survivors, additive and multiplicative models for radiation and smoking were compared, with multiplicative models providing a better fit [16]. Furthermore, in the additive model, the risk is estimated relative to nonsmokers with 0 Gy exposure, whereas in the multiplicative model, the risk is estimated relative to a participant with 0 Gy exposure with equivalent smoking status [17]. From the perspective of performance, when smoking status is not accounted for, the multiplicative model is preferred.

### Analysis models for simulation data

Analyses were conducted using Cox and Poisson regression models, with each model examined in two scenarios: including both radiation dose and smoking as covariates (Analysis 1) and including only radiation dose without adjustment for smoking (Analysis 2). The hazard functions for the Cox and Poisson regressions in Analysis 1 were described by:


$$ h(t)={h}_0(t)\exp \left({\mathrm{\beta}}_r{dose}_i+{\mathrm{\beta}}_s{smk}_i\right) $$

$$ h(t)=\exp \left({\mathrm{\beta}}_1t+{\mathrm{\beta}}_2{t}^2+{\mathrm{\beta}}_r{dose}_i+{\mathrm{\beta}}_s{smk}_i\right), $$

where ${\mathrm{\beta}}_r$ and ${\mathrm{\beta}}_s$ represent the radiation and smoking parameters, while ${\mathrm{\beta}}_1$ and ${\mathrm{\beta}}_2$ correspond to the parameters of the linear and quadratic terms of age, respectively. In the Cox regression model, ${h}_0(t)$ represented the baseline hazard that was not estimated. By contrast, Poisson regression models require an explicit estimation of baseline hazards. Therefore, age and age-squared terms were included as covariates along with radiation dose and smoking.

In the Poisson regression analyses, a person-years table was created as a preanalytical data-processing step. The number of divisions in the dose categories was varied, including values of 2, 3, 5, 30, 50, 100 and 1000, with a default of 10. Data assigned to the same group were combined into a single cell in the person-years table, accumulating the number of events and person-years. As in other software packages [18], the representative value of the radiation dose for each cell was a weighted average, with person-years serving as the weights. On the other hand, in the Cox regression analysis, no such data processing was performed, and the individual data was used directly in the analysis.

### Overall simulation procedure

The overall simulation procedure, which summarizes the conditions set up so far, is as follows.

Random numbers were generated for the time from start of follow-up to death due to cancer and due to non-cancer causes. The observed time was the shortest among these two death times and the 60-year follow-up period. Participants whose cancer-related death times were the shortest were assumed to have had events at those times, whereas those whose non-cancer-related death times or ends of their follow-up periods were the shortest were assumed to have been censored at those times. The variables available for analysis included the event or censoring time, an indicator variable indicating event or censoring, radiation dose, smoking status, and age (i.e. attained age)—with age being the only time-dependent covariate.

For the various simulation data generation settings—cohort size, radiation risk per dose, smoking risk, odds ratio of smoking probability per dose, parameters of the log-normal distribution of radiation dose and smoking probability in the control group—the results were presented so as to show how changes in the radiation parameter estimates due to the odds ratio of smoking at 1 Gy (a direct measure of the association between radiation dose and smoking) were influenced by settings other than smoking odds at 1 Gy. In each setting, the default value was selected as plausible figure, and the value was varied, including some unrealistic values, to examine the impact of each factor on the results. The default settings for the other parameters were as follows: cohort size of 80 000; a hazard ratio for cancer at 1 Gy of 1.5; a hazard ratio for smoking of 1.6; Weibull distribution shape and scale parameters for generating time of death from cancer of 6.087 and 75.99, respectively; a smoking probability of 0.3 for subjects with 0 Gy exposure; geometric mean and geometric standard deviation parameters of the log-normal distribution for radiation doses of 0.2623 and 2.223, respectively; and 10 range divisions for radiation doses when creating the person-years table. Of the various simulation data generation settings, the results are presented in terms of how the magnitude of confounding, which varies with the smoking odds at 1 Gy (i.e. the direct association between radiation and smoking), is influenced by settings other than smoking odds at 1 Gy.

The results for each setting were presented using three metrics: mean relative bias (the difference between the estimate and the true value, divided by the corresponding ERR value), mean standard error (the average standard error of the estimates), and 95% coverage probability. The number of iterations was 1000.

## RESULTS

The performances of the Cox and Poisson regression models were evaluated using two sets of covariates: smoking and radiation dose (Analysis 1) and radiation dose only (Analysis 2). These evaluations were based on the regression coefficients of radiation dose across various settings.

### Sample size of the cohort

Table 1 presents the performance evaluations of the Cox and Poisson regression models for various sample sizes.

**Table 1:** Performance evaluation of Cox and Poisson regression models with both smoking and radiation dose, and radiation dose included as covariates in terms of regression coefficients of radiation dose for various sample sizes

		Cox regression models						Poisson regression models					
		Analysis 1 (smk, dose)			Analysis 2 (dose)			Analysis 1 (smk, dose)			Analysis 2 (dose)		
Sample size	Smoking odds	Mean relative bias	Mean standard error	95% coverage probability	Mean relative bias	Mean standard error	95% coverage probability	Mean relative bias	Mean standard error	95% coverage probability	Mean relative bias	Mean standard error	95% coverage probability
20 000	0.1	−0.034	0.252	0.944	−0.594	0.253	0.918	−0.047	0.258	0.953	−0.634	0.260	0.922
20 000	0.5	−0.012	0.245	0.947	−0.244	0.245	0.946	−0.059	0.251	0.949	−0.292	0.252	0.948
20 000	1.0	−0.061	0.240	0.951	−0.073	0.240	0.948	−0.021	0.246	0.944	−0.028	0.246	0.952
20 000	2.0	−0.062	0.235	0.953	0.185	0.235	0.948	−0.066	0.241	0.953	0.190	0.241	0.939
20 000	4.0	−0.028	0.230	0.946	0.480	0.230	0.878	−0.009	0.235	0.957	0.515	0.234	0.882
20 000	8.0	−0.061	0.225	0.958	0.687	0.225	0.834	−0.018	0.230	0.944	0.758	0.228	0.793
20 000	16.0	−0.021	0.222	0.958	0.917	0.221	0.738	−0.038	0.228	0.945	0.929	0.226	0.726
20 000	32.0	−0.044	0.220	0.958	1.034	0.218	0.669	0.021	0.223	0.950	1.151	0.220	0.614
40 000	0.1	−0.013	0.177	0.953	−0.570	0.178	0.869	−0.045	0.182	0.965	−0.629	0.183	0.844
40 000	0.5	−0.019	0.172	0.952	−0.243	0.172	0.950	−0.033	0.177	0.956	−0.268	0.177	0.941
40 000	1.0	−0.012	0.169	0.934	−0.019	0.169	0.941	0.022	0.173	0.944	0.014	0.173	0.941
40 000	2.0	0.000	0.165	0.941	0.253	0.165	0.905	0.005	0.169	0.936	0.267	0.168	0.900
40 000	4.0	−0.004	0.161	0.948	0.508	0.161	0.810	−0.026	0.166	0.947	0.507	0.165	0.835
40 000	8.0	−0.022	0.159	0.952	0.724	0.158	0.697	0.016	0.161	0.950	0.793	0.160	0.650
40 000	16.0	−0.030	0.156	0.944	0.908	0.155	0.565	−0.031	0.160	0.949	0.943	0.158	0.552
40 000	32.0	−0.034	0.155	0.942	1.051	0.154	0.441	0.007	0.158	0.936	1.134	0.155	0.395
80 000	0.1	−0.023	0.125	0.962	−0.581	0.126	0.736	−0.007	0.128	0.948	−0.595	0.129	0.732
80 000	0.5	−0.030	0.122	0.957	−0.254	0.122	0.912	−0.023	0.125	0.962	−0.259	0.125	0.932
80 000	1.0	−0.013	0.119	0.952	−0.019	0.119	0.951	−0.022	0.122	0.956	−0.030	0.122	0.956
80 000	2.0	−0.005	0.116	0.945	0.244	0.116	0.904	−0.016	0.119	0.951	0.244	0.119	0.894
80 000	4.0	−0.023	0.114	0.956	0.488	0.114	0.733	−0.016	0.116	0.953	0.514	0.116	0.729
80 000	8.0	−0.021	0.112	0.944	0.726	0.112	0.483	0.004	0.114	0.942	0.780	0.113	0.453
80 000	16.0	−0.013	0.110	0.934	0.923	0.110	0.286	−0.012	0.113	0.950	0.961	0.111	0.257
**80 000**	**32.0**	**−0.014**	**0.109**	**0.941**	**1.067**	**0.108**	**0.185**	**−0.005**	**0.112**	**0.943**	**1.122**	**0.110**	**0.157**
200 000	0.1	0.002	0.079	0.957	−0.555	0.079	0.444	−0.021	0.081	0.932	−0.606	0.082	0.412
200 000	0.5	0.006	0.077	0.950	−0.219	0.077	0.860	0.016	0.079	0.948	−0.218	0.079	0.875
200 000	1.0	0.006	0.075	0.955	−0.002	0.075	0.955	−0.009	0.077	0.947	−0.015	0.077	0.944
200 000	2.0	0.008	0.074	0.953	0.256	0.074	0.799	−0.004	0.075	0.938	0.257	0.075	0.811
200 000	4.0	0.004	0.072	0.960	0.515	0.072	0.426	−0.012	0.074	0.946	0.522	0.073	0.418
200 000	8.0	0.000	0.071	0.950	0.744	0.070	0.110	−0.007	0.072	0.951	0.770	0.072	0.125
200 000	16.0	−0.010	0.070	0.959	0.924	0.069	0.021	−0.002	0.071	0.962	0.972	0.070	0.015
200 000	32.0	0.000	0.069	0.954	1.080	0.068	0.006	−0.007	0.071	0.943	1.118	0.069	0.004

Bold text indicates the representative setting.

The overall trends were similar for both regression models. In Analysis 1 (which included both smoking and radiation dose as covariates), there was minimal bias and the coverage probability was close to the nominal level of 0.95. In Analysis 2 (which included only radiation dose as a covariate), there was little bias when the smoking odds was 1.0. However, a negative bias was observed when the odds was <1.0, and a positive bias was observed when the odds was >1.0. Specifically, when the smoking odds was 32, the radiation dose estimates were biased by approximately 110%, and this bias was not affected by the sample size. In the scenarios wherein bias was observed, the coverage probability decreased as the sample size increased.

As expected, the standard errors as a whole decreased substantially as the sample size increased, and there was little difference between Analysis 1 and Analysis 2, with a slight trend toward decreasing standard errors as the odds of smoking increased. Both the Cox and Poisson regression models showed these trends, with the Poisson regression model exhibiting slightly larger standard errors (although the difference was negligible).

### ERR per Gy

Table 2 presents the performance evaluations of the Cox and Poisson regression models for various radiation risks per dose.

**Table 2:** Performance evaluation of Cox and Poisson regression models with both smoking and radiation dose, and radiation dose included as covariates in terms of regression coefficients of radiation dose for various ERR per Gy

		Cox regression models						Poisson regression models					
		Analysis 1 (smk, dose)			Analysis 2 (dose)			Analysis 1 (smk, dose)			Analysis 2 (dose)		
ERR per dose	Smoking odds	Mean relative bias	Mean standard error	95% coverage probability	Mean relative bias	Mean standard error	95% coverage probability	Mean relative bias	Mean standard error	95% coverage probability	Mean relative bias	Mean standard error	95% coverage probability
0.1	0.1	−0.120	0.131	0.942	−1.797	0.132	0.751	−0.017	0.135	0.944	−1.800	0.136	0.754
0.1	0.5	−0.023	0.128	0.951	−0.698	0.128	0.937	−0.046	0.131	0.933	−0.752	0.132	0.914
0.1	1.0	−0.073	0.125	0.962	−0.081	0.125	0.956	0.015	0.129	0.950	0.016	0.129	0.946
0.1	2.0	−0.035	0.122	0.954	0.715	0.122	0.901	−0.047	0.125	0.954	0.755	0.125	0.893
0.1	4.0	−0.012	0.119	0.955	1.534	0.119	0.749	0.012	0.123	0.961	1.633	0.122	0.718
0.1	8.0	−0.044	0.118	0.941	2.207	0.117	0.524	−0.039	0.120	0.953	2.326	0.120	0.485
0.1	16.0	−0.086	0.116	0.941	2.734	0.115	0.352	−0.077	0.118	0.955	2.876	0.117	0.299
0.1	32.0	−0.043	0.115	0.958	3.225	0.114	0.219	−0.066	0.117	0.955	3.353	0.115	0.166
0.3	0.1	−0.032	0.125	0.950	−0.590	0.125	0.723	−0.014	0.128	0.950	−0.599	0.129	0.733
0.3	0.5	−0.021	0.122	0.947	−0.246	0.122	0.917	−0.013	0.125	0.954	−0.250	0.125	0.920
0.3	1.0	−0.025	0.119	0.926	−0.033	0.119	0.928	0.013	0.122	0.952	0.005	0.122	0.955
0.3	2.0	−0.001	0.116	0.948	0.247	0.116	0.892	−0.026	0.119	0.961	0.232	0.119	0.918
0.3	4.0	−0.010	0.114	0.962	0.500	0.114	0.738	0.001	0.116	0.961	0.537	0.116	0.692
0.3	8.0	−0.011	0.112	0.942	0.736	0.112	0.485	−0.029	0.115	0.953	0.750	0.114	0.494
0.3	16.0	−0.019	0.110	0.960	0.914	0.110	0.292	0.009	0.113	0.952	0.982	0.111	0.258
**0.3**	**32.0**	**−0.004**	**0.109**	**0.956**	**1.078**	**0.108**	**0.163**	**0.003**	**0.112**	**0.947**	**1.129**	**0.110**	**0.149**
4.0	0.1	−0.001	0.086	0.942	−0.042	0.087	0.520	0.000	0.088	0.944	−0.042	0.088	0.525
4.0	0.5	0.001	0.085	0.950	−0.019	0.085	0.863	0.000	0.086	0.947	−0.021	0.086	0.852
4.0	1.0	0.001	0.083	0.949	−0.003	0.083	0.951	0.000	0.085	0.945	−0.004	0.085	0.948
4.0	2.0	−0.001	0.083	0.958	0.013	0.082	0.891	−0.001	0.084	0.951	0.014	0.083	0.892
4.0	4.0	0.000	0.081	0.947	0.035	0.081	0.576	0.000	0.082	0.943	0.035	0.082	0.595
4.0	8.0	0.000	0.080	0.946	0.052	0.080	0.271	0.000	0.081	0.961	0.053	0.081	0.249
4.0	16.0	0.001	0.080	0.955	0.067	0.079	0.082	0.001	0.081	0.947	0.069	0.080	0.074
4.0	32.0	0.001	0.079	0.951	0.078	0.078	0.024	0.000	0.080	0.947	0.079	0.079	0.031
9.0	0.1	0.000	0.076	0.949	−0.018	0.076	0.445	0.000	0.077	0.944	−0.018	0.077	0.435
9.0	0.5	0.000	0.075	0.952	−0.010	0.075	0.801	0.000	0.076	0.956	−0.010	0.076	0.800
9.0	1.0	0.001	0.075	0.954	−0.003	0.074	0.942	0.000	0.076	0.944	−0.003	0.076	0.933
9.0	2.0	0.000	0.074	0.962	0.004	0.074	0.929	0.000	0.075	0.964	0.005	0.075	0.909
9.0	4.0	0.000	0.073	0.956	0.014	0.073	0.620	0.000	0.075	0.949	0.014	0.074	0.606
9.0	8.0	0.000	0.073	0.951	0.022	0.073	0.225	0.000	0.074	0.957	0.023	0.073	0.217
9.0	16.0	0.000	0.073	0.952	0.028	0.072	0.059	0.000	0.074	0.947	0.029	0.073	0.049
9.0	32.0	0.000	0.072	0.944	0.034	0.071	0.018	0.001	0.074	0.959	0.035	0.072	0.008

Bold text indicates the representative setting.

The overall trends were similar for both regression models, with little bias observed in Analysis 1. In Analysis 2, substantial bias was observed when the ERR per Gy was set at 0.1, with the bias magnitude decreasing substantially as the ERR per Gy increased. Additionally, standard errors tended to decrease with increasing ERR per Gy. In Analysis 2, the 95% coverage probability decreased as the ERR per Gy increased, even when relative bias remained small.

### Smoking risk

Table 3 presents the performance evaluations of the Cox and Poisson regression models for various smoking risks.

**Table 3:** Performance evaluation of Cox and Poisson regression models with both smoking and radiation dose, and radiation dose included as covariates in terms of regression coefficients of radiation dose for various smoking risks

		Cox regression models						Poisson regression models					
		Analysis 1 (smk, dose)			Analysis 2 (dose)			Analysis 1 (smk, dose)			Analysis 2 (dose)		
Smoking risk	Smoking odds	Mean relative bias	Mean standard error	95% coverage probability	Mean relative bias	Mean standard error	95% coverage probability	Mean relative bias	Mean standard error	95% coverage probability	Mean relative bias	Mean standard error	95% coverage probability
1.1	0.1	−0.032	0.127	0.955	−0.136	0.127	0.950	−0.006	0.130	0.933	−0.114	0.130	0.939
1.1	0.5	0.006	0.126	0.943	−0.037	0.126	0.949	−0.016	0.130	0.942	−0.060	0.130	0.936
1.1	1.0	0.003	0.126	0.957	0.002	0.126	0.956	0.002	0.129	0.956	0.001	0.129	0.955
1.1	2.0	−0.018	0.125	0.951	0.032	0.125	0.946	−0.014	0.129	0.950	0.038	0.128	0.947
1.1	4.0	−0.004	0.125	0.940	0.099	0.125	0.928	−0.017	0.128	0.936	0.091	0.128	0.924
1.1	8.0	0.016	0.124	0.938	0.168	0.124	0.921	−0.022	0.128	0.951	0.138	0.127	0.940
1.1	16.0	−0.035	0.124	0.948	0.157	0.124	0.913	0.007	0.127	0.950	0.206	0.126	0.910
1.1	32.0	−0.001	0.124	0.951	0.226	0.123	0.906	−0.033	0.127	0.945	0.206	0.126	0.913
1.6	0.1	−0.032	0.125	0.950	−0.590	0.125	0.723	−0.014	0.128	0.950	−0.599	0.129	0.733
1.6	0.5	−0.021	0.122	0.947	−0.246	0.122	0.917	−0.013	0.125	0.954	−0.250	0.125	0.920
1.6	1.0	−0.025	0.119	0.926	−0.033	0.119	0.928	0.013	0.122	0.952	0.005	0.122	0.955
1.6	2.0	−0.001	0.116	0.948	0.247	0.116	0.892	−0.026	0.119	0.961	0.232	0.119	0.918
1.6	4.0	−0.010	0.114	0.962	0.500	0.114	0.738	0.001	0.116	0.961	0.537	0.116	0.692
1.6	8.0	−0.011	0.112	0.942	0.736	0.112	0.485	−0.029	0.115	0.953	0.750	0.114	0.494
1.6	16.0	−0.019	0.110	0.960	0.914	0.110	0.292	0.009	0.113	0.952	0.982	0.111	0.258
**1.6**	**32.0**	**−0.004**	**0.109**	**0.956**	**1.078**	**0.108**	**0.163**	**0.003**	**0.112**	**0.947**	**1.129**	**0.110**	**0.149**
5.0	0.1	−0.011	0.116	0.952	−2.200	0.118	0.000	−0.001	0.120	0.937	−2.346	0.124	0.000
5.0	0.5	−0.015	0.106	0.943	−0.908	0.106	0.258	0.005	0.109	0.954	−0.960	0.110	0.241
5.0	1.0	−0.009	0.099	0.954	−0.134	0.099	0.933	−0.029	0.102	0.938	−0.166	0.102	0.918
5.0	2.0	0.009	0.093	0.951	0.723	0.093	0.354	−0.024	0.095	0.957	0.726	0.095	0.374
5.0	4.0	−0.002	0.088	0.956	1.558	0.087	0.001	0.003	0.089	0.958	1.624	0.089	0.001
5.0	8.0	0.009	0.083	0.959	2.289	0.083	0.000	0.013	0.085	0.946	2.372	0.083	0.000
5.0	16.0	0.010	0.081	0.951	2.863	0.079	0.000	−0.014	0.082	0.954	2.941	0.080	0.000
5.0	32.0	−0.004	0.079	0.947	3.276	0.077	0.000	0.007	0.080	0.958	3.385	0.077	0.000
10.0	0.1	−0.011	0.112	0.950	−3.046	0.115	0.000	0.001	0.116	0.943	−3.276	0.122	0.000
10.0	0.5	−0.007	0.100	0.950	−1.300	0.100	0.012	0.015	0.103	0.940	−1.372	0.104	0.017
10.0	1.0	−0.024	0.093	0.952	−0.327	0.093	0.827	−0.001	0.095	0.955	−0.331	0.095	0.835
10.0	2.0	0.012	0.086	0.960	0.810	0.086	0.202	0.022	0.087	0.939	0.860	0.087	0.178
10.0	4.0	−0.016	0.080	0.954	1.887	0.080	0.000	−0.015	0.081	0.949	1.966	0.081	0.000
10.0	8.0	0.004	0.075	0.935	2.885	0.075	0.000	0.003	0.077	0.953	2.966	0.075	0.000
10.0	16.0	−0.006	0.073	0.944	3.650	0.071	0.000	0.008	0.074	0.946	3.767	0.072	0.000
10.0	32.0	0.011	0.071	0.949	4.251	0.068	0.000	−0.001	0.072	0.951	4.370	0.069	0.000

Bold text indicates the representative setting.

The overall trends were similar for both regression models. In Analysis 1, there was little bias, regardless of the smoking risk. In Analysis 2, the absolute value of the bias increased with higher smoking risk, while the standard errors decreased slightly. As the bias increased, the 95% coverage probability also decreased.

### Geometric mean of dose distribution

Table 4 presents the performance evaluations of the Cox and Poisson regression models for varying geometric means of the dose distribution.

**Table 4:** Performance evaluation of Cox and Poisson regression models with both smoking and radiation dose, and radiation dose included as covariates in terms of regression coefficients of radiation dose for various geometric means of the dose distribution

		Cox regression models						Poisson regression models					
		Analysis 1 (smk, dose)			Analysis 2 (dose)			Analysis 1 (smk, dose)			Analysis 2 (dose)		
Geometric mean	Smoking odds	Mean relative bias	Mean standard error	95% coverage probability	Mean relative bias	Mean standard error	95% coverage probability	Mean relative bias	Mean standard error	95% coverage probability	Mean relative bias	Mean standard error	95% coverage probability
0.2	0.1	−0.030	0.130	0.954	−0.587	0.130	0.730	−0.007	0.133	0.948	−0.595	0.134	0.759
0.2	0.5	−0.005	0.126	0.947	−0.229	0.126	0.935	−0.004	0.130	0.962	−0.239	0.130	0.920
0.2	1.0	0.003	0.124	0.951	−0.002	0.124	0.951	−0.035	0.128	0.951	−0.042	0.128	0.958
0.2	2.0	−0.025	0.121	0.950	0.225	0.121	0.908	−0.038	0.124	0.951	0.225	0.124	0.913
0.2	4.0	−0.017	0.119	0.949	0.497	0.119	0.743	−0.004	0.121	0.960	0.529	0.121	0.728
0.2	8.0	−0.019	0.117	0.945	0.729	0.116	0.525	−0.013	0.119	0.943	0.767	0.118	0.502
0.2	16.0	0.000	0.115	0.947	0.934	0.114	0.315	−0.002	0.117	0.957	0.971	0.116	0.310
0.2	32.0	−0.014	0.114	0.954	1.073	0.113	0.180	−0.026	0.116	0.952	1.105	0.115	0.188
0.2623	0.1	−0.022	0.125	0.957	−0.581	0.126	0.727	−0.012	0.128	0.946	−0.599	0.129	0.737
0.2623	0.5	0.001	0.121	0.956	−0.224	0.121	0.931	−0.003	0.124	0.946	−0.237	0.125	0.918
0.2623	1.0	−0.009	0.119	0.944	−0.018	0.119	0.946	−0.021	0.122	0.938	−0.029	0.122	0.943
0.2623	2.0	−0.042	0.117	0.957	0.206	0.117	0.918	−0.011	0.119	0.945	0.250	0.119	0.895
0.2623	4.0	0.001	0.114	0.953	0.510	0.114	0.724	−0.016	0.117	0.962	0.519	0.116	0.707
0.2623	8.0	−0.012	0.112	0.954	0.733	0.112	0.482	−0.019	0.114	0.963	0.759	0.114	0.470
0.2623	16.0	−0.030	0.110	0.955	0.909	0.110	0.311	−0.029	0.113	0.956	0.947	0.112	0.271
**0.2623**	**32.0**	**0.009**	**0.109**	**0.957**	**1.091**	**0.108**	**0.142**	**0.004**	**0.112**	**0.960**	**1.128**	**0.110**	**0.142**
0.8	0.1	−0.016	0.090	0.954	−0.560	0.091	0.547	−0.017	0.092	0.944	−0.580	0.093	0.535
0.8	0.5	−0.007	0.088	0.945	−0.229	0.088	0.885	−0.004	0.089	0.950	−0.235	0.090	0.894
0.8	1.0	0.000	0.086	0.934	−0.007	0.086	0.932	0.005	0.088	0.944	0.000	0.088	0.942
0.8	2.0	−0.004	0.084	0.956	0.245	0.084	0.847	−0.003	0.086	0.956	0.253	0.085	0.844
0.8	4.0	0.002	0.082	0.959	0.511	0.082	0.541	−0.016	0.084	0.941	0.513	0.083	0.544
0.8	8.0	0.002	0.081	0.945	0.745	0.080	0.219	−0.003	0.082	0.955	0.764	0.081	0.207
0.8	16.0	−0.007	0.080	0.960	0.921	0.079	0.070	−0.002	0.081	0.952	0.953	0.080	0.064
0.8	32.0	−0.008	0.079	0.944	1.058	0.078	0.023	0.015	0.080	0.948	1.111	0.079	0.016
1.5	0.1	0.007	0.062	0.948	−0.517	0.062	0.297	0.004	0.063	0.945	−0.534	0.063	0.258
1.5	0.5	−0.011	0.060	0.949	−0.233	0.060	0.785	−0.014	0.061	0.946	−0.242	0.061	0.780
1.5	1.0	−0.022	0.059	0.939	−0.029	0.059	0.943	0.000	0.059	0.946	−0.007	0.059	0.942
1.5	2.0	−0.007	0.057	0.956	0.244	0.057	0.724	0.002	0.058	0.940	0.259	0.058	0.707
1.5	4.0	−0.004	0.056	0.952	0.508	0.056	0.229	−0.002	0.057	0.949	0.523	0.056	0.219
1.5	8.0	−0.005	0.055	0.939	0.737	0.055	0.025	−0.010	0.056	0.927	0.746	0.055	0.028
1.5	16.0	−0.002	0.055	0.957	0.916	0.054	0.000	−0.005	0.055	0.956	0.932	0.054	0.001
1.5	32.0	−0.003	0.054	0.938	1.045	0.053	0.000	0.005	0.055	0.945	1.070	0.053	0.000

Bold text indicates the representative setting.

The overall trends were similar for both regression models, with minimal bias in Analysis 1. However, Analysis 2 revealed bias caused by changes in smoking odds—although the trend remained largely unaffected by the geometric mean of the dose distribution. In both analyses, the standard error decreased as the geometric mean increased. In Analysis 1, the 95% coverage probability remained constant, whereas in Analysis 2 (which showed bias), the coverage probability decreased as the standard error decreased.

### Geometric standard deviation of dose distribution

Table 5 presents the performance evaluations of the Cox and Poisson regression models for various geometric standard deviations of the dose distribution.

**Table 5:** Performance evaluation of Cox and Poisson regression models with both smoking and radiation dose, and radiation dose included as covariates in terms of regression coefficients of radiation dose for various geometric standard deviations of the dose distribution

		Cox regression models						Poisson regression models					
		Analysis 1 (smk, dose)			Analysis 2 (dose)			Analysis 1 (smk, dose)			Analysis 2 (dose)		
Geometric standard deviation	Smoking odds	Mean relative bias	Mean standard error	95% coverage probability	Mean relative bias	Mean standard error	95% coverage probability	Mean relative bias	Mean standard error	95% coverage probability	Mean relative bias	Mean standard error	95% coverage probability
1	0.1	−0.062	2.757	0.951	−0.816	2.757	0.948	−0.912	3.802	0.947	−35.463	3.991	0.950
1	0.5	0.141	2.754	0.963	−0.113	2.754	0.965	−0.603	3.596	0.950	−1.059	3.600	0.947
1	1.0	−0.259	2.748	0.950	−0.199	2.748	0.953	−0.323	3.588	0.941	−0.276	3.589	0.934
1	2.0	−0.422	2.754	0.947	−0.170	2.754	0.949	−0.469	3.608	0.951	−0.050	3.604	0.950
1	4.0	−0.238	2.747	0.954	0.241	2.746	0.950	−0.404	3.591	0.958	0.427	3.585	0.957
1	8.0	−0.001	2.742	0.952	0.730	2.742	0.949	−0.939	3.621	0.946	0.291	3.607	0.952
1	16.0	−0.423	2.748	0.956	0.555	2.748	0.957	0.488	3.597	0.945	2.235	3.584	0.937
1	32.0	−0.012	2.744	0.948	1.279	2.744	0.944	0.152	3.569	0.944	2.123	3.553	0.943
2	0.1	−0.017	0.188	0.943	−0.604	0.189	0.851	−0.073	0.198	0.958	−0.721	0.200	0.820
2	0.5	−0.013	0.184	0.951	−0.242	0.184	0.945	−0.008	0.192	0.960	−0.252	0.193	0.948
2	1.0	−0.040	0.181	0.944	−0.048	0.181	0.946	−0.026	0.189	0.956	−0.035	0.189	0.957
2	2.0	0.008	0.177	0.937	0.256	0.177	0.904	−0.014	0.184	0.954	0.253	0.184	0.909
2	4.0	−0.022	0.174	0.944	0.488	0.174	0.853	−0.022	0.181	0.952	0.534	0.180	0.835
2	8.0	−0.022	0.170	0.957	0.730	0.170	0.712	−0.003	0.177	0.951	0.807	0.176	0.706
2	16.0	−0.049	0.169	0.951	0.904	0.168	0.604	0.004	0.175	0.948	1.029	0.173	0.540
2	32.0	−0.018	0.167	0.969	1.101	0.165	0.492	0.010	0.173	0.956	1.211	0.170	0.414
2.223	0.1	−0.022	0.125	0.957	−0.581	0.126	0.727	−0.012	0.128	0.946	−0.599	0.129	0.737
2.223	0.5	0.001	0.121	0.956	−0.224	0.121	0.931	−0.003	0.124	0.946	−0.237	0.125	0.918
2.223	1.0	−0.009	0.119	0.944	−0.018	0.119	0.946	−0.021	0.122	0.938	−0.029	0.122	0.943
2.223	2.0	−0.042	0.117	0.957	0.206	0.117	0.918	−0.011	0.119	0.945	0.250	0.119	0.895
2.223	4.0	0.001	0.114	0.953	0.510	0.114	0.724	−0.016	0.117	0.962	0.519	0.116	0.707
2.223	8.0	−0.012	0.112	0.954	0.733	0.112	0.482	−0.019	0.114	0.963	0.759	0.114	0.470
2.223	16.0	−0.030	0.110	0.955	0.909	0.110	0.311	−0.029	0.113	0.956	0.947	0.112	0.271
**2.223**	**32.0**	**0.009**	**0.109**	**0.957**	**1.091**	**0.108**	**0.142**	**0.004**	**0.112**	**0.960**	**1.128**	**0.110**	**0.142**
3	0.1	0.004	0.052	0.950	−0.497	0.052	0.180	0.000	0.053	0.948	−0.506	0.053	0.167
3	0.5	0.004	0.051	0.961	−0.217	0.051	0.749	−0.008	0.051	0.960	−0.230	0.051	0.738
3	1.0	−0.008	0.049	0.957	−0.015	0.049	0.955	−0.009	0.050	0.951	−0.016	0.050	0.948
3	2.0	−0.002	0.048	0.946	0.250	0.048	0.645	0.003	0.049	0.944	0.256	0.048	0.641
3	4.0	−0.011	0.047	0.953	0.503	0.047	0.113	−0.002	0.047	0.954	0.516	0.047	0.096
3	8.0	0.001	0.046	0.957	0.735	0.046	0.005	0.005	0.047	0.942	0.749	0.046	0.001
3	16.0	0.002	0.046	0.951	0.901	0.045	0.001	−0.002	0.046	0.951	0.907	0.045	0.000
3	32.0	−0.006	0.046	0.950	1.004	0.045	0.000	−0.009	0.046	0.943	1.014	0.045	0.000

Bold text indicates the representative setting.

When the geometric standard deviation was 1, both regression models produced unstable results, with some bias in Analysis 1 and an irregular bias change in Analysis 2. In this scenario, the standard error increased substantially for both regression models, although the Cox regression model consistently showed smaller standard errors compared to the Poisson one.

When the geometric standard deviation differed from 1, there was little difference between the two models, and the pattern of bias change with smoking odds was nearly as regular as it was in other settings. As the geometric standard deviation increased, the absolute value of the bias decreased in Analysis 2, and the overall standard errors tended to decrease.

### Baseline smoking probability

Table 6 presents the performance evaluations of the Cox and Poisson regression models for various baseline smoking probabilities.

**Table 6:** Performance evaluation of Cox and Poisson regression models with both smoking and radiation dose, and radiation doses included as covariates in terms of regression coefficients of radiation dose for various baseline smoking probability

		Cox regression models						Poisson regression models					
		Analysis 1 (smk, dose)			Analysis 2 (dose)			Analysis 1 (smk, dose)			Analysis 2 (dose)		
Baseline smoking probability	Smoking odds	Mean relative bias	Mean standard error	95% coverage probability	Mean relative bias	Mean standard error	95% coverage probability	Mean relative bias	Mean standard error	95% coverage probability	Mean relative bias	Mean standard error	95% coverage probability
0.1	0.1	−0.011	0.127	0.957	−0.224	0.127	0.947	0.004	0.130	0.963	−0.219	0.130	0.932
0.1	0.5	−0.015	0.126	0.956	−0.109	0.126	0.958	−0.030	0.129	0.950	−0.131	0.129	0.947
0.1	1.0	−0.021	0.125	0.961	−0.024	0.125	0.956	−0.030	0.128	0.945	−0.033	0.128	0.950
0.1	2.0	0.004	0.123	0.957	0.132	0.123	0.939	−0.013	0.126	0.956	0.125	0.126	0.938
0.1	4.0	−0.023	0.121	0.945	0.292	0.121	0.867	−0.012	0.124	0.932	0.317	0.124	0.870
0.1	8.0	−0.026	0.119	0.952	0.513	0.119	0.732	−0.013	0.121	0.955	0.553	0.121	0.710
0.1	16.0	−0.028	0.117	0.952	0.750	0.117	0.504	−0.024	0.119	0.945	0.793	0.118	0.463
0.1	32.0	−0.006	0.114	0.954	0.996	0.114	0.269	0.003	0.116	0.949	1.043	0.116	0.251
0.3	0.1	−0.003	0.125	0.951	−0.561	0.125	0.753	−0.046	0.129	0.959	−0.465	0.130	0.834
0.3	0.5	0.013	0.121	0.957	−0.211	0.121	0.929	0.006	0.127	0.950	−0.175	0.127	0.936
0.3	1.0	−0.027	0.119	0.949	−0.035	0.119	0.947	−0.027	0.125	0.944	−0.032	0.125	0.941
0.3	2.0	0.009	0.116	0.941	0.256	0.116	0.883	0.007	0.122	0.947	0.228	0.122	0.897
0.3	4.0	−0.006	0.114	0.955	0.503	0.114	0.715	−0.014	0.120	0.949	0.464	0.119	0.764
0.3	8.0	−0.007	0.112	0.949	0.741	0.111	0.481	−0.012	0.117	0.955	0.725	0.116	0.524
0.3	16.0	0.005	0.110	0.953	0.940	0.110	0.279	−0.020	0.115	0.955	0.955	0.114	0.303
**0.3**	**32.0**	**−0.012**	**0.109**	**0.952**	**1.070**	**0.108**	**0.184**	**0.006**	**0.113**	**0.944**	**1.164**	**0.112**	**0.143**
0.5	0.1	−0.003	0.122	0.950	−0.775	0.123	0.524	−0.009	0.126	0.962	−0.827	0.127	0.493
0.5	0.5	−0.026	0.117	0.957	−0.301	0.117	0.899	0.001	0.120	0.944	−0.288	0.120	0.907
0.5	1.0	−0.014	0.115	0.953	−0.021	0.115	0.953	−0.020	0.117	0.944	−0.026	0.117	0.941
0.5	2.0	−0.012	0.112	0.953	0.236	0.112	0.904	−0.024	0.115	0.939	0.239	0.115	0.893
0.5	4.0	−0.007	0.110	0.962	0.458	0.110	0.743	−0.010	0.113	0.952	0.475	0.112	0.744
0.5	8.0	0.011	0.108	0.956	0.648	0.108	0.546	−0.005	0.111	0.951	0.657	0.110	0.553
0.5	16.0	−0.026	0.108	0.944	0.739	0.107	0.451	0.001	0.110	0.952	0.795	0.109	0.406
0.5	32.0	0.010	0.107	0.956	0.867	0.106	0.298	0.001	0.109	0.954	0.892	0.108	0.305
0.7	0.1	−0.005	0.119	0.955	−0.823	0.118	0.456	0.005	0.122	0.949	−0.857	0.122	0.454
0.7	0.5	−0.033	0.113	0.950	−0.273	0.113	0.909	0.001	0.115	0.952	−0.254	0.115	0.917
0.7	1.0	−0.028	0.111	0.942	−0.034	0.111	0.948	−0.007	0.113	0.961	−0.012	0.113	0.959
0.7	2.0	−0.019	0.109	0.947	0.162	0.108	0.918	−0.009	0.111	0.938	0.181	0.111	0.912
0.7	4.0	0.003	0.107	0.949	0.324	0.107	0.830	−0.005	0.110	0.925	0.329	0.110	0.832
0.7	8.0	0.018	0.107	0.951	0.438	0.106	0.761	−0.017	0.109	0.952	0.418	0.108	0.767
0.7	16.0	−0.011	0.106	0.946	0.482	0.106	0.705	−0.037	0.109	0.948	0.476	0.108	0.730
0.7	32.0	−0.012	0.106	0.934	0.532	0.105	0.655	0.009	0.108	0.950	0.576	0.107	0.637

Bold text indicates the representative setting.

The overall trends were similar for both regression models, with minimal bias observed in Analysis 1. When the smoking odds were > 1, Analysis 2 showed a positive bias—with the highest absolute bias occurring at a baseline smoking probability of 0.3 and decreasing trends for both smaller and larger probabilities. Conversely, when the smoking odds were < 1, Analysis 2 showed a negative bias, with the absolute value of the bias increasing as the baseline smoking probability increased. Standard errors remained constant across both analyses and all baseline smoking probability settings, whereas 95% coverage probabilities varied with the magnitude of bias.

### Number of categories in the person-years table

Table 7 presents the performance evaluations of the Poisson regression models for various baseline smoking probabilities.

**Table 7:** Performance evaluation of Poisson regression models with both smoking and radiation dose, and radiation doses included as covariates in terms of regression coefficients of radiation dose for various numbers of categories in the person-years table

		Poisson regression models												
		Analysis 1 (smk, dose)			Analysis 2 (dose)				Analysis 1 (smk, dose)			Analysis 2 (dose)		
Smoking odds	Categories in person-years tables	Mean relative bias	Mean standard error	95% coverage probability	Mean relative bias	Mean standard error	95% coverage probability	Categories in person-years table	Mean relative bias	Mean standard error	95% coverage probability	Mean relative bias	Mean standard error	95% coverage probability
0.1	2	−0.003	0.156	0.938	−0.860	0.162	0.658	15	−0.013	0.127	0.955	−0.585	0.127	0.732
0.5	2	−0.021	0.151	0.946	−0.360	0.153	0.895	15	0.015	0.123	0.940	−0.216	0.123	0.927
1.0	2	−0.008	0.148	0.946	−0.021	0.148	0.945	15	−0.008	0.121	0.957	−0.017	0.121	0.956
2.0	2	0.027	0.142	0.943	0.392	0.141	0.854	15	−0.013	0.118	0.947	0.242	0.118	0.893
4.0	2	−0.005	0.139	0.940	0.736	0.137	0.627	15	−0.001	0.115	0.942	0.523	0.115	0.715
8.0	2	−0.025	0.136	0.956	1.034	0.132	0.352	15	−0.037	0.113	0.953	0.725	0.113	0.505
16.0	2	−0.005	0.134	0.947	1.307	0.128	0.147	15	0.001	0.112	0.944	0.956	0.111	0.279
32.0	2	−0.010	0.133	0.944	1.492	0.126	0.060	15	−0.020	0.111	0.932	1.085	0.109	0.175
0.1	3	−0.044	0.142	0.944	−0.755	0.145	0.670	30	−0.014	0.126	0.954	−0.578	0.126	0.724
0.5	3	−0.013	0.137	0.947	−0.294	0.138	0.914	30	−0.012	0.122	0.947	−0.238	0.122	0.932
1.0	3	−0.016	0.134	0.959	−0.023	0.134	0.953	30	−0.019	0.120	0.938	−0.026	0.120	0.944
2.0	3	0.001	0.131	0.948	0.310	0.130	0.878	30	−0.014	0.117	0.956	0.236	0.117	0.911
4.0	3	−0.003	0.127	0.960	0.625	0.126	0.684	30	−0.002	0.114	0.958	0.511	0.114	0.707
8.0	3	0.011	0.124	0.937	0.921	0.122	0.373	30	−0.020	0.113	0.949	0.731	0.112	0.494
16.0	3	−0.009	0.123	0.939	1.127	0.120	0.212	30	−0.018	0.111	0.950	0.924	0.110	0.287
32.0	3	−0.016	0.122	0.947	1.288	0.117	0.112	30	−0.025	0.110	0.948	1.065	0.109	0.178
0.1	5	−0.028	0.133	0.950	−0.660	0.135	0.701	100	−0.021	0.125	0.937	−0.577	0.125	0.728
0.5	5	−0.025	0.130	0.945	−0.278	0.130	0.914	100	−0.035	0.122	0.957	−0.261	0.122	0.921
1.0	5	0.015	0.126	0.956	0.006	0.126	0.951	100	−0.004	0.119	0.957	−0.012	0.119	0.953
2.0	5	0.002	0.124	0.950	0.281	0.123	0.887	100	0.006	0.116	0.947	0.255	0.116	0.886
4.0	5	−0.015	0.121	0.948	0.556	0.120	0.687	100	−0.018	0.114	0.965	0.498	0.114	0.722
8.0	5	−0.020	0.118	0.953	0.805	0.117	0.471	100	−0.020	0.112	0.947	0.726	0.112	0.498
16.0	5	−0.013	0.116	0.946	1.022	0.114	0.222	100	−0.012	0.110	0.952	0.927	0.110	0.297
32.0	5	−0.002	0.115	0.968	1.190	0.112	0.127	100	−0.007	0.109	0.941	1.078	0.108	0.183
0.1	10	−0.007	0.128	0.956	−0.594	0.129	0.740	1000	−0.034	0.125	0.951	−0.591	0.126	0.737
0.5	10	−0.012	0.125	0.948	−0.247	0.125	0.912	1000	−0.010	0.122	0.955	−0.235	0.122	0.923
1.0	10	−0.021	0.122	0.940	−0.028	0.122	0.942	1000	0.010	0.119	0.958	0.000	0.119	0.955
2.0	10	−0.014	0.119	0.939	0.248	0.119	0.890	1000	−0.034	0.117	0.947	0.219	0.117	0.911
4.0	10	−0.013	0.117	0.956	0.521	0.116	0.709	1000	−0.010	0.114	0.957	0.499	0.114	0.708
8.0	10	−0.007	0.114	0.947	0.767	0.113	0.457	1000	−0.017	0.112	0.945	0.727	0.112	0.492
16.0	10	−0.007	0.112	0.937	0.969	0.111	0.273	1000	0.002	0.110	0.940	0.936	0.109	0.264
**32.0**	**10**	**−0.023**	**0.112**	**0.951**	**1.103**	**0.110**	**0.165**	1000	−0.010	0.109	0.957	1.073	0.108	0.164

Bold text indicates the representative setting.

Under all settings, Analysis 1 showed minimal bias. Analysis 2 exhibited a bias for settings with smoking odds other than 1.0; however, the absolute value of this bias decreased as the number of categories in the person-years table increased.

### Heterogeneity in baseline risk

Table 8 presents the performance evaluations of the Cox and Poisson regression models for various magnitudes of heterogeneity in baseline risk.

**Table 8:** Performance evaluation of Cox and Poisson regression models with both smoking and radiation dose, and radiation dose included as covariates in terms of regression coefficients of radiation dose for various magnitude of heterogeneity in baseline risk

		Cox regression models						Poisson regression models					
		Analysis 1 (smk, dose)			Analysis 2 (dose)			Analysis 1 (smk, dose)			Analysis 2 (dose)		
Baseline risk coefficient	Smoking odds	Mean relative bias	Mean standard error	95% coverage probability	Mean relative bias	Mean standard error	95% coverage probability	Mean relative bias	Mean standard error	95% coverage probability	Mean relative bias	Mean standard error	95% coverage probability
0.0	0.1	−0.032	0.125	0.950	−0.590	0.125	0.723	−0.014	0.128	0.950	−0.599	0.129	0.733
0.0	0.5	−0.021	0.122	0.947	−0.246	0.122	0.917	−0.013	0.125	0.954	−0.250	0.125	0.920
0.0	1.0	−0.025	0.119	0.926	−0.033	0.119	0.928	0.013	0.122	0.952	0.005	0.122	0.955
0.0	2.0	−0.001	0.116	0.948	0.247	0.116	0.892	−0.026	0.119	0.961	0.232	0.119	0.918
0.0	4.0	−0.010	0.114	0.962	0.500	0.114	0.738	0.001	0.116	0.961	0.537	0.116	0.692
0.0	8.0	−0.011	0.112	0.942	0.736	0.112	0.485	−0.029	0.115	0.953	0.750	0.114	0.494
0.0	16.0	−0.019	0.110	0.960	0.914	0.110	0.292	0.009	0.113	0.952	0.982	0.111	0.258
**0.0**	**32.0**	**−0.004**	**0.109**	**0.956**	**1.078**	**0.108**	**0.163**	**0.003**	**0.112**	**0.947**	**1.129**	**0.110**	**0.149**
0.1	0.1	0.000	0.124	0.953	−0.557	0.125	0.757	−0.015	0.128	0.949	−0.599	0.129	0.735
0.1	0.5	−0.010	0.121	0.940	−0.238	0.121	0.903	−0.007	0.124	0.946	−0.241	0.125	0.910
0.1	1.0	−0.013	0.119	0.956	−0.021	0.119	0.953	−0.032	0.122	0.944	−0.039	0.122	0.939
0.1	2.0	−0.003	0.116	0.949	0.244	0.116	0.892	0.003	0.119	0.961	0.263	0.119	0.892
0.1	4.0	0.006	0.114	0.953	0.515	0.114	0.687	−0.026	0.116	0.963	0.511	0.116	0.717
0.1	8.0	−0.008	0.112	0.951	0.741	0.111	0.491	−0.025	0.114	0.953	0.752	0.113	0.480
0.1	16.0	−0.007	0.110	0.952	0.925	0.109	0.287	−0.016	0.113	0.952	0.955	0.111	0.263
0.1	32.0	−0.011	0.109	0.952	1.069	0.108	0.176	−0.009	0.111	0.941	1.115	0.110	0.155
0.5	0.1	−0.059	0.121	0.949	−0.592	0.121	0.707	−0.029	0.124	0.954	−0.593	0.124	0.723
0.5	0.5	−0.050	0.118	0.956	−0.267	0.118	0.912	−0.068	0.121	0.954	−0.294	0.121	0.905
0.5	1.0	−0.029	0.115	0.964	−0.036	0.115	0.960	−0.036	0.118	0.949	−0.043	0.118	0.948
0.5	2.0	−0.031	0.113	0.953	0.209	0.113	0.897	−0.015	0.115	0.955	0.234	0.115	0.907
0.5	4.0	−0.055	0.111	0.949	0.437	0.111	0.763	−0.040	0.113	0.952	0.474	0.113	0.749
0.5	8.0	−0.045	0.109	0.958	0.673	0.108	0.531	−0.062	0.111	0.945	0.688	0.110	0.539
0.5	16.0	−0.045	0.107	0.945	0.856	0.107	0.333	−0.041	0.110	0.949	0.896	0.108	0.288
0.5	32.0	−0.040	0.106	0.939	1.001	0.105	0.197	−0.026	0.109	0.948	1.059	0.107	0.168
1.0	0.1	−0.140	0.112	0.947	−0.596	0.112	0.651	−0.168	0.115	0.928	−0.647	0.116	0.630
1.0	0.5	−0.157	0.110	0.940	−0.342	0.110	0.862	−0.143	0.113	0.942	−0.335	0.113	0.886
1.0	1.0	−0.145	0.108	0.930	−0.151	0.108	0.924	−0.160	0.111	0.937	−0.165	0.111	0.937
1.0	2.0	−0.160	0.107	0.929	0.048	0.107	0.940	−0.133	0.109	0.956	0.083	0.109	0.945
1.0	4.0	−0.150	0.105	0.943	0.276	0.104	0.878	−0.147	0.107	0.943	0.301	0.107	0.862
1.0	8.0	−0.168	0.103	0.925	0.459	0.103	0.726	−0.171	0.106	0.932	0.481	0.105	0.717
1.0	16.0	−0.161	0.102	0.914	0.629	0.101	0.525	−0.146	0.104	0.936	0.676	0.103	0.492
1.0	32.0	−0.179	0.101	0.921	0.735	0.100	0.408	−0.169	0.104	0.941	0.784	0.102	0.379

Bold text indicates the representative setting.

In both Analysis 1 and Analysis 2, as the heterogeneity of baseline risk increased, there was an overall bias toward underestimation. This trend was observed in both the Cox and Poisson regression models. In settings where the smoking odds were < 1, and underestimation had been observed in other results, this underestimation became more pronounced. Conversely, in settings where the smoking odds were > 1 and overestimation had been observed in other results, part of this overestimation was offset by underestimation, resulting in a reduced bias.

## DISCUSSION

In this study, we quantitatively assessed the confounding effects of smoking on radiation risk estimates in a cohort of radiation workers using simulations. Given the wide variability present in previous epidemiological study, we simplified the situation to fit it into the framework of a simulation study. We selected a setting where the smoking odds was set at 32 (see ‘Relationship between smoking and radiation dose’ subsection below for details), with other factors set to their default values as a representative setting. We then quantified the effect of confounding factors of smoking as the odds of smoking per dose, summarizing how this value changed with other factors.

In this representative setting, the results from Analysis 1, which was adjusted for smoking, showed minimum bias. Conversely, in Analysis 2, which did not adjust for smoking, the risk was overestimated by approximately 110% (For example, in Table 2, the mean bias in Analysis 1 was 1.078, compared with almost 0 in Analysis 2.) The magnitude of this bias depended on ERR per Gy, smoking risk, baseline smoking probability, heterogeneity in baseline risk, and the odds of smoking per dose—which reflected the strength of the relationship between smoking and radiation dose. However, the bias was largely independent of cohort size, dose distribution parameters, and number of categories in the person-years table of Poisson regression models.

With an increase in ERR per Gy, the relative bias, which is divided by the corresponding ERR value, tended to decrease substantially (Table 2). However, in the settings where the smoking odds deviated from 1, the 95% coverage probability decreased, suggesting that the bias appears smaller only superficially. The heterogeneity of baseline risk led to underestimation (Table 8), known as ‘omitted covariates’—omission of covariates that are associated with outcome but not associated with other covariates. Although some research has been conducted on this topic [19–21], it will not be further addressed in this study. The results also varied with the geometric mean and geometric standard deviation of the dose distribution, which were likely influenced by the stability of the estimation (Tables 4 and 5).

Adjusting for smoking was shown to change radiation risk estimates in the Japanese epidemiological study on low-dose radiation effects. The ERR per Sv for all cancers, excluding leukemia, decreased from 0.80 (90% CL: −0.39, 2.19) to 0.29 (90% CL: −0.81, 1.57) after adjusting for smoking—indicating a 176% overestimation if the smoking-adjusted estimate is accurate [6]. Compared to the 110% overestimation derived from this study, the overestimation estimated from the real-world J-EPISODE epidemiological study was higher. As demonstrated by the results (Table 2), where the ERR per Gy was varied, as the true ERR per Gy decreased, the absolute values of both overestimation and underestimation increased sharply. Considering that these findings and the estimate from the J-EPISODE study were as low as 0.29 and had a wide confidence interval, the results of our simulation were consistent with those of actual epidemiological studies.

### Simulation settings

In previous epidemiological studies on radiation, participants have typically been chronically exposed to radiation and continue to be exposed throughout the follow-up periods of the studies. In this study, we assumed that the participants had already received any type of radiation exposure by the start of the follow-up period. As a result, cumulative doses may have been overestimated at the beginning of the follow-up period compared to those in previous real-world epidemiological studies. However, our results suggest that confounding effects are largely independent of dose distribution, which should have little impact on the conclusions of this study. There is generally no increase in radiation dose exposure after such workers retire because they are no longer exposed to the radiation from their workplace. This therefore aligns with our simulation settings concerning postretirement radiation workers.

Radiation effects are known to be modified by age at exposure and attained age. Some studies have evaluated smoking cumulatively, as pack-years, and examined its interaction with radiation dose [16, 17]. Longitudinal patterns must also be considered when dealing with radiation and smoking over time; however, data for such settings are limited. In this study, radiation was treated as a single exposure and smoking as a binary variable. We modeled the association between radiation and smoking as an odds ratio of the probability of smoking per dose. This simplification allows us to express confounding as a function of an easily interpretable measure—smoking odds—and to identify how these changes are modified by other factors.

It is also known that the effects of radiation vary depending on the dose rate, and the risk of developing solid cancers is often lower with low-dose-rate exposure compared to that observed with high-dose-rate, for example, as observed in atomic bomb survivors [22]. Furthermore, in long-term follow-up scenarios such as those involving radiation workers, it is necessary to adjust for radiation sensitivity because age changes over the follow-up period [23]. Because this study focused on the confounding effects of smoking and radiation, these effects were not considered in addition to various factors described above. It will be necessary to conduct further investigations into the confounding effects of smoking and radiation, taking into account time-dependent cumulative dose and dose-rate effectiveness factor by referencing findings from various studies.

### Relationship between smoking and radiation dose

Several epidemiological studies of radiation workers have described an association between radiation dose and smoking. While some studies did not show a clear correlation [24, 25], others did [26, 27], particularly a study with Japanese cohort [28].

In the above-mentioned Japanese cohort study, a graph showing the smoking proportion by age and dose category indicated that, among individuals in their 30s (i.e. a moderate sample size that was less affected by saturation), approximately 63% of those in the group who were exposed to radiation levels of <10 mSv smoked, compared to 78% in the group who were exposed to >100 mSv. This resulted in an odds ratio of smoking of 2.08 between these two groups. Assuming that the average dose for the >100 mSv group was 200 mSv (which was not stated in the reference), the odds of smoking in the <10 mSv and 1 Sv groups would have been 39.14. Although the results can vary substantially depending on the assumed average dose in the >100 mSv group, the setting of 32 for the smoking odds in this study would be considered plausible.

### Limitations

This study quantified the confounding effects of smoking in a model analysis by comparing results with and without adjustments for smoking. The analysis in the present study assumed that the risks of radiation and smoking were modeled correctly. Incorrect modeling can introduce numerous other biases that are too varied to specifically address. Although robust methods exist for model misspecifications, they are beyond the scope of the present study.

Occupational cohorts are generally healthier than the general population because of the initial health selection often required for employment. This is known as the healthy worker effect [27–29]. Therefore, analyses estimating the radiation-related risk in such cohorts rely primarily on internal comparisons and are sometimes adjust for employment duration. Adjustments are often made because healthier individuals tend to remain employed for longer periods and thus accumulate higher radiation doses. However, employment duration can also be a part of the outcome (i.e. the time to event onset), and adjusting for outcome-related variables may introduce bias [30]. This issue is complex, not only in the current analysis but also when simulating the healthy worker effect, as there are many possible settings through which health status can be incorporated into the scenario. As a result, we did not address this issue in the present study.

In conclusion, we conducted a simulation study to quantitatively evaluate the bias from confounding and modeling conditions, similar to actual epidemiological studies. Our analysis, based on data from Japanese radiation workers, indicated that not adjusting for smoking can lead to an overestimation of radiation effects by approximately 110%. This overestimation was relatively insensitive to sample size and dose distribution parameters—but varied with radiation and smoking risk, baseline smoking probability, and heterogeneity in baseline risk. Considering the simplified settings of this simulation study and the uncertainty of the estimates of Japanese radiation workers, our simulation results aligned with those of the real-world epidemiological study.
